# Response of Balanced Laminate Subjected to Abrupt Heat Flux: A New Perspective

**DOI:** 10.1155/2022/9491939

**Published:** 2022-04-14

**Authors:** Salih N. Akour

**Affiliations:** Department of Mechanical Engineering, School of Engineering, The University of Jordan, Amman 11942, Jordan

## Abstract

A new perspective presents how the interaction between the material stiffness of both matrix and fibre took place to form the laminate response. Thin square simply supported balanced laminated composite plate of different ply arrangements subjected to abrupt heat flux is utilized in this investigation. The effect of ply arrangement on the elapse time for vibrations to vanish and the maximum response amplitude is also investigated. The finite element analysis results show that the response is an amplitude modulated signal that represents frequencies of both fibre and matrix. The analysis shows that the ratio of the carrier frequency to the modulating frequency matches the ratio of the fibre stiffness to ply lateral stiffness. Through the investigation, the maximum static deformation and the dynamic amplitude are illustrated for different ply arrangements. Furthermore, it is found that almost all laminates have a similar elapsed time for vibrations to vanish.

## 1. Introduction

Vibration response and natural frequencies of structures are very important issues for designers to know. Composite laminate plates are one of these structures and their importance is highly increased in the last decade. Many structures such as aerospace vehicles, airplanes, and automotive vehicles are made of composite laminated plates. This topic received the attention of many researchers worldwide. The research on composite plates is carried out either analytically, numerically, or both.

Analytical solution for laminated plates is well established for special cases in reference books such as in reference [1]. In these cases, the coupling between extensional stiffness and bending stiffness does not exist. The equations (1) and (2) show the general formulation of the composite laminate whereas equation (3) presents the simplified form of equation (2) when *B*_*ij*_ = 0, *A*_16_ = *A*_26_ = *D*_16_ = *D*_26_ = 0, i.e., coupling is eliminated. In such a special case, a closed form for first natural frequency for a square plate of side length *a* can be obtained as shown in equation (4) [1].

(1) $$\begin{array}{c} \left[\begin{array}{c} N_{x}\\ N_{y}\\ N_{xy} \end{array}\right]=\left[\begin{array}{ccc} A_{11} & A_{12} & A_{16}\\ A_{12} & A_{22} & A_{26}\\ A_{16} & A_{26} & A_{66} \end{array}\right]\cdot \left[\begin{array}{c} \varepsilon_{x}^{0}\\ \varepsilon_{y}^{0}\\ \gamma_{xy}^{0} \end{array}\right]+\left[\begin{array}{ccc} B_{11} & B_{12} & B_{16}\\ B_{12} & B_{22} & B_{26}\\ B_{16} & B_{26} & B_{66} \end{array}\right]\cdot \left[\begin{array}{c} k_{x}\\ k_{y}\\ k_{xy} \end{array}\right],\\ \left[\begin{array}{c} M_{x}\\ M_{y}\\ M_{xy} \end{array}\right]=\left[\begin{array}{ccc} B_{11} & B_{12} & B_{16}\\ B_{12} & B_{22} & B_{26}\\ B_{16} & B_{26} & B_{66} \end{array}\right]\cdot \left[\begin{array}{c} \varepsilon_{x}^{0}\\ \varepsilon_{y}^{0}\\ \gamma_{xy}^{0} \end{array}\right]+\left[\begin{array}{ccc} D_{11} & D_{12} & D_{16}\\ D_{12} & D_{22} & D_{26}\\ D_{16} & D_{26} & D_{66} \end{array}\right]\cdot \left[\begin{array}{c} k_{x}\\ k_{y}\\ k_{xy} \end{array}\right], \end{array}$$

(2) $$\begin{array}{c} D_{11}\frac{\partial^{4}w}{\partial x^{4}}+4D_{16}\frac{\partial^{4}w}{\partial x^{3}\partial y}+2\left(D_{12}+2D_{66}\right)\frac{\partial^{4}w}{\partial x^{2}\partial y^{2}}+4D_{26}\frac{\partial^{4}w}{\partial y^{3}\partial x}+D_{22}\frac{\partial^{4}w}{\partial y^{4}}\\ -B_{11}\frac{\partial^{3}u^{0}}{\partial x^{3}}-3B_{16}\frac{\partial^{3}u^{0}}{\partial x^{2}\partial y}-\left(B_{12}+2B_{66}\right)\frac{\partial^{3}u^{0}}{\partial x\;\partial y^{2}}-B_{26}\frac{\partial^{3}u^{0}}{\partial y^{3}}-B_{16}\frac{\partial^{3}v^{0}}{\partial x^{3}}\\ -\left(B_{12}+2B_{66}\right)\frac{\partial^{3}v^{0}}{\partial y\;\partial x^{2}}-3B_{26}\frac{\partial^{3}v^{0}}{\partial x\;\partial y^{2}}-B_{22}\frac{\partial^{3}v^{0}}{\partial y^{3}}+\rho h\frac{\partial^{2}w^{0}}{\partial t^{2}}=q_{T}\left(x,y\right), \end{array}$$

(3) $$\begin{array}{c} D_{11}\frac{\partial_{4}w}{\partial x^{4}}+2\left(D_{12}+2D_{66}\right)\frac{\partial^{4}w}{\partial x^{2}\partial y^{2}}+D_{22}\frac{\partial^{4}w}{\partial y^{4}}+\rho h\frac{\partial^{2}w}{\partial t^{2}}=0, \end{array}$$

(4) $$\begin{array}{c} \omega_{11}^{2}=\frac{\pi^{4}}{\rho_{0}.a^{4}}\left[D_{11}+2\left(D_{12}+2D_{66}\right)+D_{22}\right]. \end{array}$$

Examples from the literature for obtaining the natural frequencies of composite laminates using analytical solutions can be found in references [2–4]. The researchers are exploiting either the refined shear deformation theory or higher-order refined theory, respectively, to catch as much as they can from the problem physics.

In references [5, 6], the researchers utilized numerical methods in modelling and analysing the vibrations of laminated composite plates. They either use commercial software packages and/or develop their own code in their investigations.

Some other researchers use both analytical and numerical analysis in studying the vibrations of composite laminates such as in references [7–11]. They utilized different plate theories along with numerical analysis in conducting their study.

In the current study, the applied loading is sudden heat flux as excitation. This type of loading ends up as an impact bending moment load acting at the boundary of the laminate plate. As composite structures are broadly utilized in the aeronautic industry, recently, thermally induced vibration investigations of laminated composite plates turn out to be increasingly significant. As an example, space vehicle usually has large elastic and bendable members that carry solar panels. These are connected to its main body. Its reliability decides whether the space station functions successfully and securely. It is subjected to several thermal loads such as solar irradiance while orbiting around the Earth with a space station. The thermal distortion of such a structure happens due to the abrupt exposure of the solar panel assembly to the solar irradiance while it is circling around the earth. There were numerous publications by scientists in this direction. Some of these are going to be surveyed in the following paragraphs.

Thornton and Kim [12] investigated thermally induced structural dynamics of the elastic and bendable appendages on a satellite that commonly happens during eclipse alterations. They developed a numerical approach for the thermal-structural coupling analysis of tensegrity structures considering the prestressed state after the form-finding process. Johnston and Thornton [13] elaborated on the effect of the thermal-structural performance of deployable appendages on satellite attitude dynamics and control. Thermally induced structural movements of appendages such as solar panels and booms grow the load on the main body of a satellite. The performance of these appendages was investigated analytically and experimentally. The model was integrated into a coupled dynamic modelling for the planar dynamics of a simple satellite comprising a bendable appendage and a rigid hub. Yang et al. [14] considered the combined conduction and radiation heat transfer in a foldable solar panel of space vehicles in orbit. They considered the thermal effect on the temperature response of the foldable solar panel. Li et al. [15] analysed the nonlinear dynamics of practical lean‐walled big‐scale space structures subjected to abrupt heat load. The coupling consequence between structural distortions and the incident direction of solar heat flux was considered. Results verified the necessary condition of vibrations that are induced by thermal heat load and proved the principle of thermal flutter. Kim and Han [16] investigated a thorough thermal model for fixed-type solar panels in a low-earth orbit satellite both analytically and numerically. Under the worst hot scenario, analytical results showed that the solar reflector type had the least temperature. Akour [17] investigated laminated plates exposed to rapid surface heating. Boundary conditions applied along the four edges of the laminate were simple support. Utilizing finite element analysis, the effect of plate depth and ply arrangement on the maximum deformation was studied. The investigation presented that overlooking the coupling between the induced moments and in-plan loads in the antisymmetric cross-ply laminate although it is small in comparison with the other coefficients will deliver deceptive results.

This investigation sheds a light on how the stiffness materials comprising the laminate interact to produce the response of the composite laminate. A balanced laminate plate is investigated to present such interaction. The current investigation is considering the dynamic response of thin balanced laminate plates of different ply arrangements subjected to abrupt or sudden heat flux. A comparison between cross-ply laminates, unidirectional, and angled ply laminates is investigated to find the elapsed time needed for the oscillation to vanish.

## 2. Numerical Technique

The thin square laminated composite plate is modelled utilizing finite element method (FEM) and using (T300/5208) composite material according to the following configurations: unidirectional (0/0/0/0), symmetric cross-ply (0/90/90/0), antisymmetric cross-ply (0/90/0/90), symmetric angle-ply (30/−30/−30/30), antisymmetric angle-ply (30/−30/30/−30), symmetric angle-ply (45/−45/−45/45), and antisymmetric angle-ply (45/−45/45/−45).

The plate is modelled as a thin plate, i.e., the thickness to side length ratio is 0.05. The laminates are simply supported along the four edges. ANSYS Commercial Package Software is used to perform all FEM analyses regarding all cases.

Graphite epoxy laminate T300/5208 as a composite material is exploited in this research.  Table 1 displays the material properties utilized in the investigation [1]. The chosen material for the investigation is broadly employed in the industry.

The thermally induced vibration in laminates represents a thermal-structure coupled problem. The detailed treatments for the coupled problem approach can be found in reference [17].

### 2.1. Thermal Model

Element shell-132 is used in developing the thermal FEM model of the laminate. This type of element introduces the heat flux as a face load. The model is built as a square area and meshed using square elements of equal-size. The mesh is refined until the difference between the successive iterations is less than 1%. The model is abruptly exposed to an even constant thermal heat flux over its top surface at time zero. While the bottom surface is perfectly insulated. Under this condition, i.e., there is no temperature variation that takes place in the x-y plane. The only temperature variation that takes place is in the *z*-direction.

### 2.2. Structural Model

Element shell-99 is utilized to model the laminates with simply supported boundary conditions for all sides of the laminates. The plate structure is made of multiple plies that are assumed to perfectly adhere to each other so that the structure acts as a nonuniform anisotropic plate. Based on the perfect adherence assumption, no-slip occurs between the plies, and continuous displacements are assumed through the plies.

#### 2.2.1. Laminate Structure Analysis

The following subsections explain the different stacking sequence arrangements and the coupling between in-plane and out-of-plane stiffness coupling.

#### 2.2.2. Symmetric and Unidirectional Laminates

There are no couplings between the extensional and bending stiffness, as shown in Table 2.

#### 2.2.3. The Antisymmetric Cross-Ply Laminate

It has a coupling stiffness in *B*_11_ and *B*_22_. This explains the different behaviour of the antisymmetric cross-ply laminate deformation compared to the other laminates.

#### 2.2.4. The Symmetric Laminate

No coupling exists between the extensional and the bending stiffness. Therefore, the generated bending moment is a result of the heat flux, which goes mainly as bending deformation. This explains why the symmetric laminates have high deformation values compared with the others.

#### 2.2.5. Antisymmetric Angle Ply Laminates

**Figure 1:**
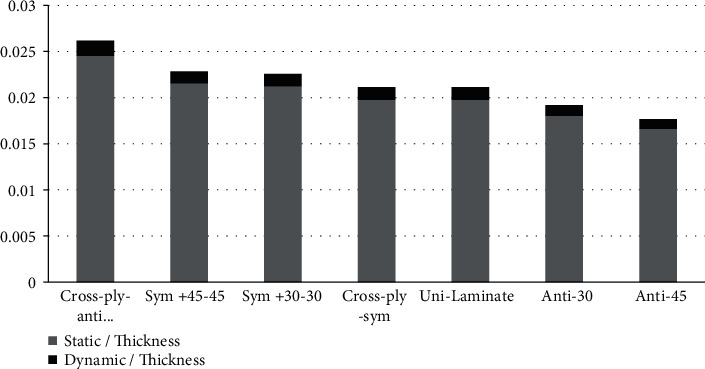
Presentation of the static amplitude of the laminate response shown in pattern fill and the maximum dynamic amplitude shown in black solid color.

These laminates have stiffness coupling at *B*_16_ and *B*_26_ and have the least deformation as is obvious from Figure 1. This coupling between the extensional and bending deformation leads to low out-of-plane deformation. The affected terms by this coupling are shown in equations (1) and (2). Consequently, the input energy comes out as a work done by both bending moment and extension forces. This eliminates completely the coupling, so the analytical solution can be obtained. This kills part of the problem physics when the coupling does exist and is ignored. The first natural frequency shown in equation (4) is for this special case of the square plate of side length *a* [1].

## 3. Results and Discussion

Based on references [18, 19], only the first mode shape for the deformation profile of the plates is considered in this investigation because it is the main mode shape, and it represents a maximum displacement. Moreover, it is well known that for a simply supported plate, the maximum deformation arises when the first mode shape occurs rather than the other mode shapes for a similar amount of input energy.

**Figure 2:**
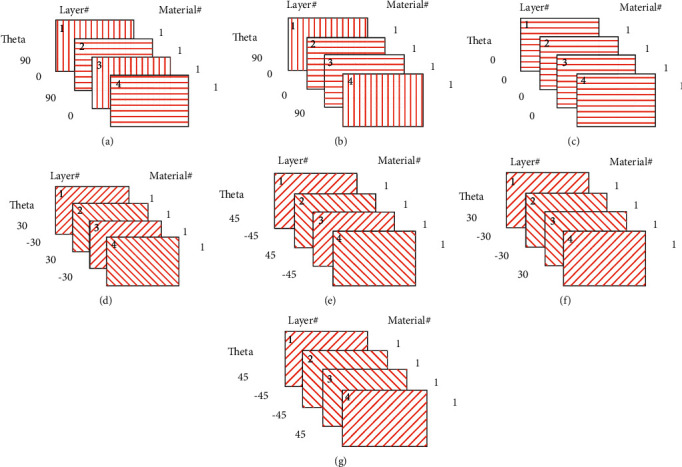
Illustration of the laminate ply arrangement: (a) antisymmetric cross-ply, (b) symmetric cross-ply, (c) unidirectional, (d) antisymmetric 30, (e) antisymmetric 45, (f) symmetric 30, and (g) symmetric 45.

Figure 2 illustrates the ply arrangement for the different types of laminates that are used in this investigation. This ply arrangement is recognized as a balanced laminate.

The material properties are independent of the temperature only over a relatively small temperature range; attention is restricted here to the response within the time range of 0 < *t* < 60 seconds [19]. Within this time extent, the temperature increase is below 100°C.

**Figure 3:**
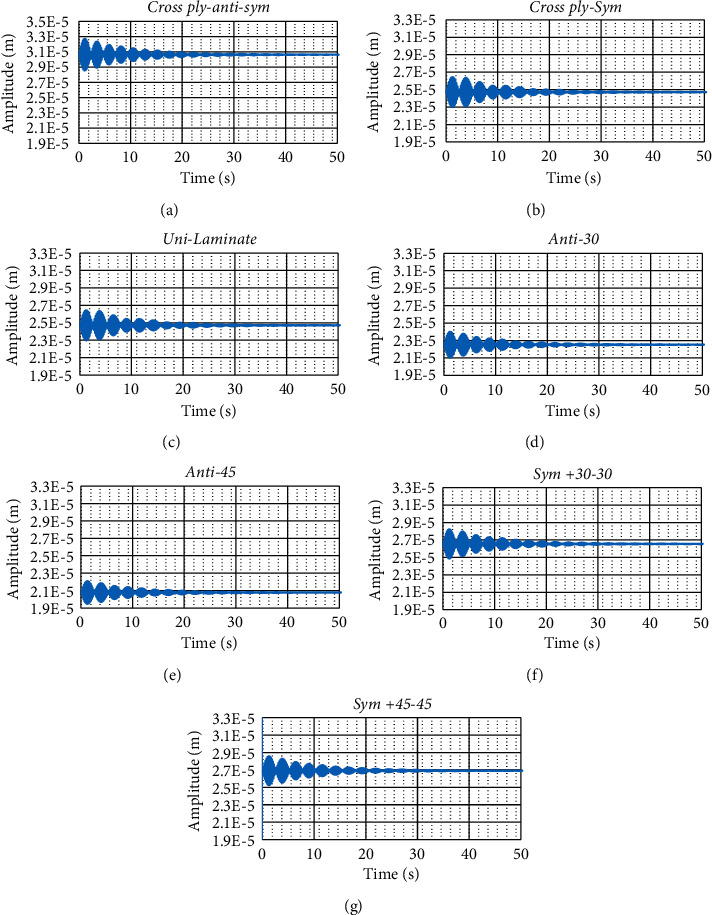
Illustration of the response of the laminates for the abrupt heat flux in all graphs.

**Figure 4:**
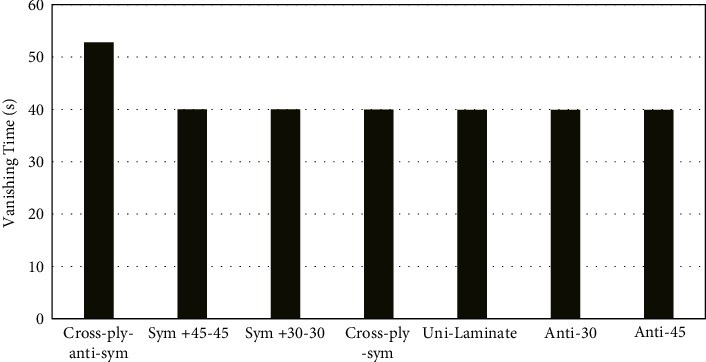
Presentation of the time elapsed till the vibration vanishes.

The dynamic responses for all laminates are presented in Figure 3. For all laminates, the decay of the vibration amplitude with time is almost the same for all cases. The antisymmetric cross-ply laminate has maximum static deformation (average amplitude) compared to the rest laminates. Figure 1 shows both the static amplitude and the dynamic amplitude. The antisymmetric ply laminates have the largest total deformation (both the static and dynamic amplitudes), whereas the unidirectional laminate has the least total deformation. The elapsed time for vanishing the oscillation of the laminates is shown in Figure 4. All the laminates have almost the same vanishing time, except the anti-cross-ply laminate is a little bit larger than the others.

**Figure 5:**
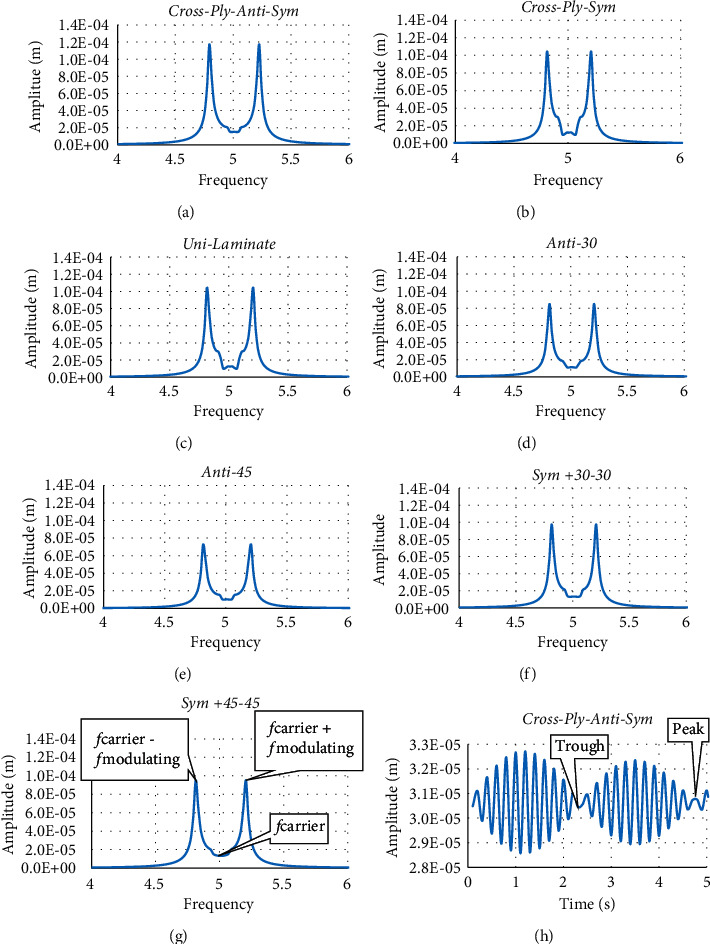
Illustration of the Fourier analysis for the output response of the laminates. The Sym + 45 − 45 laminate graph shows both carrier and modulating frequencies. The last graph is an enlarged sample of response for cross-ply antisymmetric laminate that shows the trend of amplitude modulation.

It is obvious from Figure 3 that the response for all cases is amplitude modulated. The amplitude modulation usually occurs when there is a carrier signal modulated by another one, i.e., from a mathematical point of view, it is the multiplication of the carrier signal and modulating signal. The response of all laminate cases under investigation are subjected to Fourier analysis to obtain the frequency content of the response signal as shown in Figure 5(a)through 5(g). All cases have modulating frequency of 0.196 and carrier frequency of 5. The modulating frequency can be obtained from the graphs as half the distance between the two peaks as illustrated in Figure 5(g). As it can be seen that the carrier amplitude is low compared to the modulating amplitude. This is what causes the wave to flip in direction as it can be seen in Figure 5(h) where at the end of the first half of the signal there is a trough and at the end, there is a peak.

Analysis of the amplitude modulation shows that the ratio of the carrier frequency with respect to the modulating frequency is 25. By comparing the ratio of fibre modulus of elasticity *E*_*f*_ with respect to the ply lateral modulus of elasticity *E*_2_, it can be found that *E*_*f*_/*E*_2_ ratio is 22.76. This ratio almost matches the carrier and the modulating frequency ratio which is 25. The difference between the two ratios is less than 9%. Since the laminate comprises two different materials that make one structure, fibre impeded in an epoxy matrix, each material will vibrate according to its stiffness which is mainly controlled by its modulus of elasticity. According to Figure 1, the laminates have static deformation and dynamic deformation. Due to the static deformation, the fibres impeded inside the matrix are experiencing tension and vibrating according to its stiffness (i.e., modulus of elasticity). Due to the static deformation, most of the load will be carried as tension by the fibres. Since the fibre stiffness is high, then, the natural frequency of the fibre is high compared to the matrix material's natural frequency. Along the fibre direction, the fibre stiffness is dominant whereas in the lateral direction *E*_2_, the matrix material properties are dominant. Since the matrix is attached to the fibre, the lateral modulus of elasticity of the matrix is improved to be *E*_2_, i.e., the fibre does not carry any load in the lateral direction but improves the modulus of elasticity of the matrix through the stickiness of the matrix particles on the fibres.

## 4. Conclusions and Future Work

A study is conducted for exploring the response of laminated composite plate subjected to sudden heat flux. Based on the obtained numerical results for thin plates of symmetric, antisymmetric, and unidirectional laminates, the following conclusions can be drawn:

- The response of the laminates is amplitude modulation which represents the natural frequency of the materials (components) forming the laminate, i.e., fibre and matrix. This will provide designers with better knowledge on how to design the structure when applying the excitation load frequency.
- All laminates except the antisymmetric cross-ply laminate have a similar elapse time for vibrations to vanish. This makes the antisymmetric cross-ply laminate the least candidate as a choice for designers.
- Cross-ply antisymmetric laminate has shown the largest response compared to the others. The symmetric laminates and unidirectional laminates come next in the amount of response amplitude.
- The antisymmetric angled ply laminates showed the least amount of response amplitude. This makes it a better choice for designers.

Further work is necessary to generalize the study for a wider range of ply stacking, different types of loading, and different types of boundary conditions.

## Figures and Tables

**Table 1:** Properties of material used in the investigation [1].

Property	Item	T300/5208	Unit
Thermal	Density	1600	kg/m^3^
	Specific heat	760	(J/K° kg)
	Thermal conductivity *x*-direction	0.72	W/(m K°)

Fibre properties	Modulus of elasticity	230	GPa
	Fibre volume fraction	0.785	
	Poisson's ratio	0.22	

Epoxy properties	Modulus of elasticity	2.25	GPa
	Matrix volume fraction	0.215	
	Poisson's ratio	0.38	

Structural	Modulus of elasticity in the *x*-direction (*E*_1_)	181	GPa
	Modulus of elasticity in the *y*-direction (*E*_2_)	10.1	GPa
	Coefficient of thermal expansion *x*-direction	3 ∗ 10^−7^	1/K°
	Coefficient of thermal expansion *y*-direction	2.5 ∗ 10^−5^	1/K°
	Shear modulus	7.2	GPa

Applied load	Abrupt heat flux	1370	W/m^2^

**Table 2:** Summary of the ply arrangement cases.

Ply arrangement name	Ply arrangement	Stiffness coefficients
Cross-ply anti-sym	[90°/0°/90°/0°]	*B*_22_ ≠ 0, *B*_11_ ≠ 0, *B*_16_=0, *B*_26_=0, *B*_12_=0, *B*_66_=0, *D*_11_ ≠ 0, *D*_12_ ≠ 0, *D*_22_ ≠ 0, *D*_66_ ≠ 0, *D*_16_=*D*_26_=0
Cross-ply sym	[90°/0°/0°/90°]	*B*_*ij*_=0, *D*_11_ ≠ 0, *D*_12_ ≠ 0, *D*_22_ ≠ 0, *D*_66_ ≠ 0, *D*_16_=*D*_26_=0
Uni-laminate	[0°/0°/0°/0°]	*B*_*ij*_=0, *D*_11_ ≠ 0, *D*_12_ ≠ 0, *D*_22_ ≠ 0, *D*_66_ ≠ 0, *D*_16_=*D*_26_=0
Anti-30	[30°/−30°/30°/−30°]	*B*_16_ ≠ 0, *B*_26_ ≠ 0, *B*_12_=0, *B*_22_=0, *B*_11_=0, *B*_66_=0, *D*_11_ ≠ 0, *D*_12_ ≠ 0, *D*_22_ ≠ 0, *D*_66_ ≠ 0, *D*_16_=*D*_26_=0
Anti-45	[45°/−45°/45°/−45°]	*B*_16_ ≠ 0, *B*_26_ ≠ 0, *B*_12_=0, *B*_22_=0, *B*_11_=0, *B*_66_=0, *D*_11_ ≠ 0, *D*_12_ ≠ 0, *D*_22_ ≠ 0, *D*_66_ ≠ 0, *D*_16_=*D*_26_=0
Sym +30 − 30	[30°/−30°/−30°/30°]	*B*_*ij*_=0, *D*_*ij*_ ≠ 0
Sym +45 − 45	[45°/−45°/−45°/45°]	*B*_*ij*_=0, *D*_*ij*_ ≠ 0s

## Data Availability

The data used to support the findings of this study are included within the article.
